# Production and Antibacterial Activity of Atypical Siderophore from *Pseudomonas* sp. QCS59 Recovered from *Harpachene schimperi*

**DOI:** 10.3390/ph17091126

**Published:** 2024-08-26

**Authors:** Mashael A. Almuhawish, Essam Kotb, Eida Alkhaldi, Asmaa A. Ahmed

**Affiliations:** 1 Basic and Applied Scientific Research Center (BASRC), Imam Abdulrahman Bin Faisal University (IAU), P.O. Box 1982, Dammam 31441, Saudi Arabia; malalmuhwish@iau.edu.sa; 2 Department of Biology, College of Science, Imam Abdulrahman Bin Faisal University (IAU), P.O. Box 1982, Dammam 31441, Saudi Arabia; ealkhaldi@iau.edu.sa; 3 Department of Statistics, Faculty of Commerce, Al-Azhar University, Cairo P.O. Box 11751, Egypt; asmaaahmed.2055@azhar.edu.eg

**Keywords:** siderophore, wastewater flora, iron, antibacterial, *Pseudomonas*

## Abstract

Among sixty-eight pseudomonads, isolate QCS59 from the rhizosphere of *H. schimperi* was selected based on its siderophore level. Production was optimal in Kings B supplemented with 2% peptone and 0.5% fructose at pH 6.5 and 25 °C for 72 h. Additionally, the threshold potential of iron was found at a concentration of 10 µM. After purification, the acidified siderophore presented a maximum absorption peak of 360 nm, while the neutral form presented a maximum of 414 nm, confirming its pyoverdine (PVD) nature. Furthermore, a major peak appeared at a retention time (RT) of 27.5 min during RP-HPLC, confirming its homogeneity. Interestingly, it demonstrated effective antibacterial activity, especially against *Escherichia coli* ATCC 8739, with a minimum inhibitory concentration (MIC) of 6.3 µg/mL and a minimum bactericidal concentration (MBC) of 12.5 µg/mL. At ½ the MIC value, it inhibited 82.1% of well-established biofilms of *Salmonella enterica*. There was an increase in malondialdehyde (MDA) and antioxidative enzymes, especially catalase (CAT) in the treated bacteria because of the peroxidation of membrane lipids and oxidative stress, respectively. SEM proved cellular lysis and surface malformation in most of the treated bacteria. This study concludes that QCS59 siderophore is a promising antibacterial candidate for treating wastewater bacteria and skin pathogens.

## 1. Introduction

Iron is the most plentiful mineral in the environment and is crucial for all living organisms, including microorganisms, because of its involvement in various physiological pathways, such as photosynthesis, oxidative phosphorylation, the tricarboxylic acid cycle, and the electron transport chain. However, under aerobic conditions, the free form of iron (Fe^3+^) forms insoluble structures of hydroxides and oxyhydroxides, leading to reductions in its availability and absorption by living organisms [1]. Bacteria can solubilize these insoluble iron structures via the secretion of small-size chelating agents, termed siderophores. Based on their structure, they are categorized into catecholate, hydroxamate, phenolate, carboxylate, and mixed types of siderophores [2,3].

Studies indicated that Gram-positive and negative bacteria can produce them in iron-depleted environmental conditions [4]. Upon the secretion of siderophores outside of cells, they conjugate with iron and other minerals, such as Zn, Al, Pb, Cu, Cd, Ga, and In, with different affinities based on the kind of metal, type of siderophore, and its necessity for the producing bacterium [1,5]. There are about five hundred siderophores, which contain a large number of regulators and genes responsible for biosynthesis, transportation, and the entry of minerals into cells [6].

Siderophores bind ferric iron and form siderophore–iron conjugates that re-enter bacterial cells via given receptors located on the cell. In Gram-negative bacteria, the passage machinery is complicated owing to the complicated membrane organization. The siderophore–iron complex transfers to the periplasm through a periplasmic binding protein communicating with an ATP-binding cassette transporter. In Gram-positive bacteria, the movement of siderophore–iron conjugates is quite different and is only conducted by permeases, ATPases, and irrevocable siderophore proteins [3,7]. Once the conjugate arrives inside the cytosol, Fe^3+^ ions are reduced to Fe^2+^ ions and are released from the composite for use in different physiological processes occurring inside bacterial cells, while the released siderophore is either reprocessed or damaged by the flow from the efflux pumps [8].

The continuing antibiotic resistance crisis and the horizontal gene transfer of resistance elements among bacteria inside the human body, animal body, wastewater, and environment demand new antibacterial agents other than antibiotics to treat and prevent these multidrug-resistant bacteria [9]. The introduction of new antibiotics has been disappointing because resistance ultimately develops within a few years; in addition, antibiotics themselves end up in the environment, causing selective pressure on indigenous bacteria that convert them into antibiotic-resistant forms [2]. Numerous investigators have described alternatives, including siderophores, as inhibitors for pathogenic microbes [1,10]; however, no iron-blockade option has been effectively developed to treat wastewater bacteria [11].

Several members of genus *Pseudomonas*, such as *P. fluorescens*, *P. aeruginosa*, *P. syringae*, and *P. putida*, can secrete many types of siderophores; in addition, many fungi, algae, and other bacteria, can produce them. They have secondary functions for the producing microorganisms besides the acquisition of iron; for example, xenosiderophores can inhibit the growth of surrounding microorganisms. This property can make them good alternatives to traditional drugs. It was found that the siderophore of *Burkholderia paludis* greatly inhibited the growth of many enterococci and staphylococci [3]. Furthermore, the siderophore of *P. aeruginosa* Mgrv7 greatly affected *Candida* spp., especially *C. tropicalis* and *C. albicans* [10], and the siderophores of *P. putida* showed an outstanding inhibitory action against several fungi [12]. In addition, the siderophores of *P. fluorescens* BBc6R8 showed biocontrol activity against the bacterium *Streptomyces ambofaciens* ATCC23877 [13].

To counteract the effect of xenosiderophores, microorganisms create a defensive antioxidative system involving the superoxide dismutase (SOD) enzyme, which can breakdown superoxides into H_2_O_2_, which is further hydrolyzed into H_2_O and O_2_ by CAT [14]. Unfortunately, the overproduction of ROS negatively affects the morphological structure of bacteria as it frequently results in peroxidation and disorganization of cell membrane lipids, thus resulting in growth inhibition and death [15,16]. This study aimed to perform the first screening for siderophore-producing pseudomonads in the environment in the eastern region of Saudi Arabia. We characterized the siderophore production of a newly isolated *Pseudomonas* strain sp. QCS59, inspected its physical tolerance, and explored its antibacterial activity against many pathogenic bacteria, especially water-borne flora.

## 2. Results

### 2.1. Screening for Siderophore-Producing Bacteria

**Figure 1:**
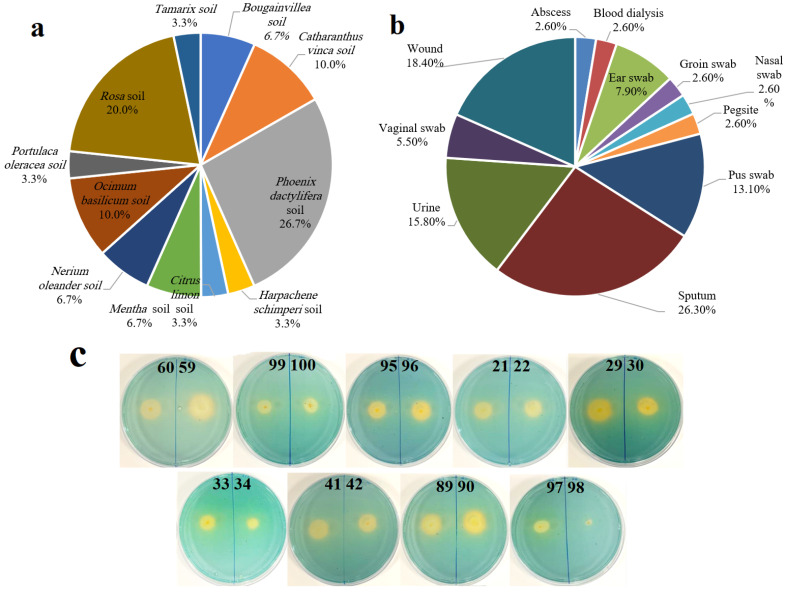
Percentage of recovered siderophore-producing *Pseudomonas* isolates by source plant (**a**) and source clinical sample (**b**). Samples were gathered from several areas of the eastern region of Saudi Arabia, then bacteria were isolated on the specific medium cetrimide agar with incubation at 30 °C for 48 h to allow the growth of suspected pseudomonads. Qualitative universal CAS assay (**c**) for siderophore production by *Pseudomonas* isolates was performed by spotting actively growing bacterial isolates on CAS agar and incubating at 25 °C for 48 h. The transformation of blue reagent into orange color around bacterial isolates is an indication of siderophore production.

Out of the examined soil samples, thirty isolates of *Pseudomonas* were recovered on cetrimide agar. Sample No. 27, collected from Khobar, Saudi Arabia, from the rhizosphere of *Phoenix dactylifera*, was the superior sample, with eight *Pseudomonas* isolates recovered (Figure 1a), followed by a sample collected from the rhizospheric region of *Rosa* (six isolates were obtained). In parallel, a total of thirty-eight clinical pseudomonads were recovered from different specimens such as sputum, wound, urine, pus swab, ear swab, abscess, dialysis blood, groin swab, nasal swab, pegsite, and vaginal swab specimens. The largest numbers of clinical *Pseudomonas* spp. were recovered from sputum samples (26.3%), followed by wounds (18.4%) (Figure 1b).

Bacteria isolated on cetrimide agar were spotted on Chrome Azurol Sulfonate (CAS) reagent agar to evaluate siderophore production. The highest level of siderophore was obtained from isolate No. 59; therefore, this isolate was selected for further investigations. Figure 1c shows the conversion of the faint blue color of CAS reagent into yellow to light orange color after 24 h of incubation, confirming siderophore production due to the reaction of siderophore with reagent.

**Figure 2:**
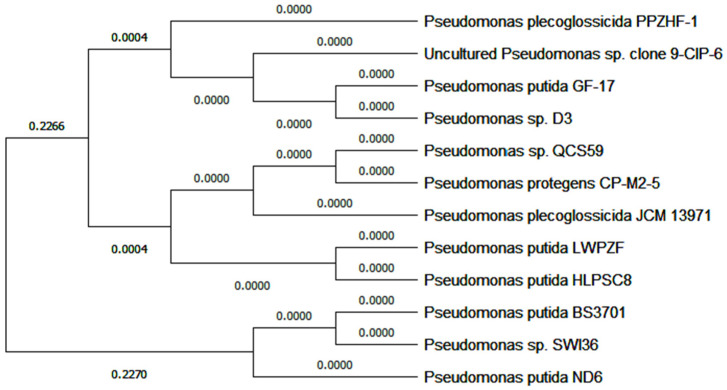
Phylogenetic tree generated with MEGA11 software for isolate number 59. It was identified genetically by sequencing of the *16SrDNA* gene after DNA amplification with PCR using 1492R primer and 27F primer. The BLAST search tool was applied to search for homologous sequences, then the phylogeny was constructed as a neighbor-joining tree. The 0.0000 values of most branches (horizontal lines) represent the amount of genetic change (genetic distance) between strains over time. The internal nodes represent putative ancestors for the sampled strains. For this, the QCS59 isolate’s sequence was 100% similar to many species of genus *Pseudomonas* such as *putida* and *protegens* and was designated as sp. The tree is rooted in the ultimate common ancestor of all aligned strains. Ancestor and time run from left to right, which is referred to as a molecular clock. The numbers near nodes are generally at 0–1. Values near 1 mean that there is a strong indication that the sequences to the right of the node cluster together to the exclusion of any other.

Microbiologically, the selected isolate was found to be an obligate aerobe producing translucent entire colonies, which appeared as Gram-negative rods under light microscopy. It was β-hemolytic, flagellated, uncapsulated, and non-lactose fermenter. It was positive on oxidase, CAT, and citrate utilization tests while negative on indole, methyl red, and Voges-Proskauer tests. Molecular characterization was performed based on *16SrDNA* gene sequence analysis. Homologous sequences available in the GenBank database were established via BLAST analysis, and the selected isolate was deposited in the database (accession number: MT153203). The blasted sequence showed 100% similarity to *P. protegens* CP-M2-5 (accession number: MT790722.1), *P. plecoglossicida* JCM 13971 (accession number: LC507999.1), and other species such as *P. putida;* for this, no specific species was assigned at the species level. The phylogenetic tree generated by Molecular Evolutionary Genetics Analysis version 11 (MEGA11) software is shown in Figure 2, and strain code QCS59 was assigned.

### 2.2. Optimization of Nutritional and Physical Conditions Affecting Siderophore Production by Pseudomonas *sp.* QCS59

**Figure 3:**
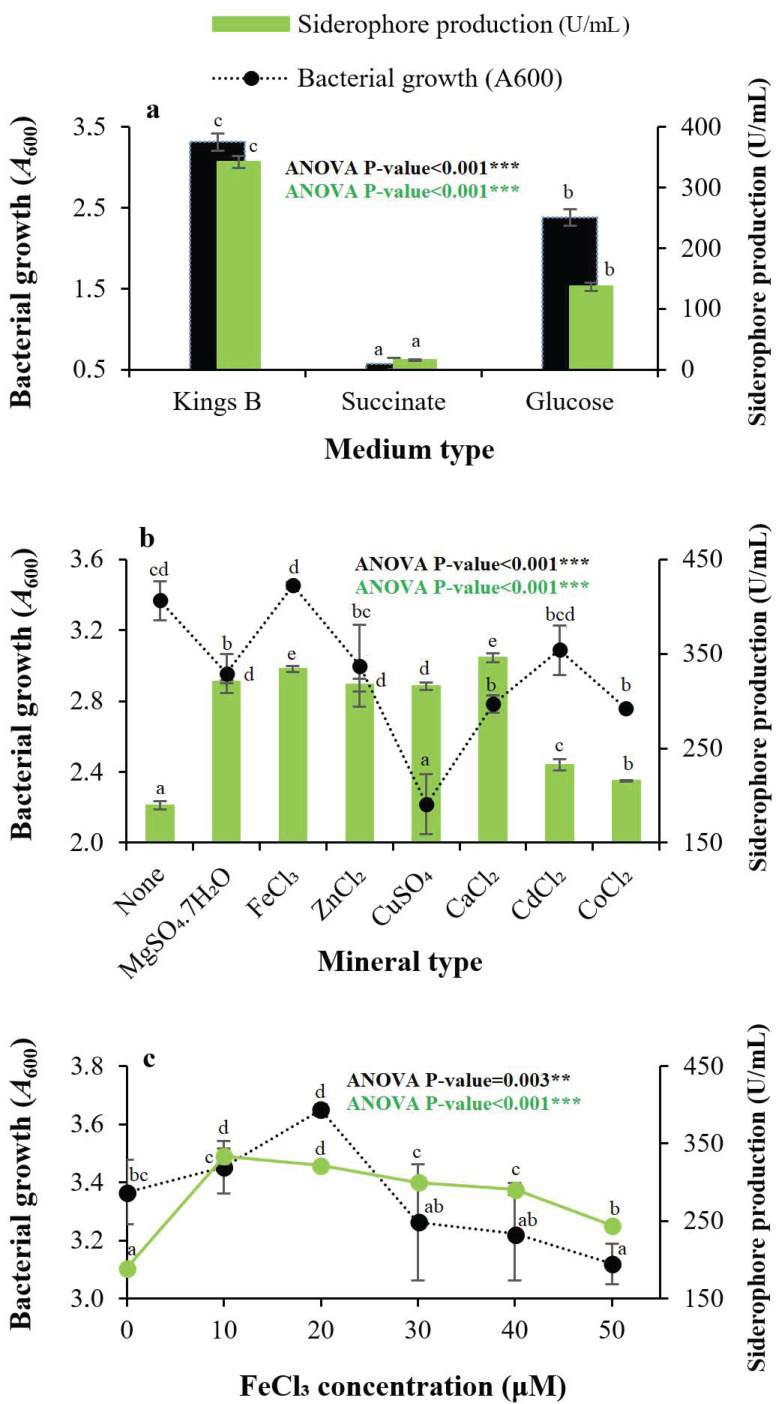
Effect of medium type on growth and siderophore production by *Pseudomonas* sp. QCS59 (**a**). Incubation was performed at pH 7.0, 25 °C, 150 rpm for 72 h. Uninoculated media were used as control. The effect of common mineral salts at 20.0 μM concentration was determined separately to determine the optimal minerals for production (**b**). Effect of FeCl_3_ concentration on siderophore production and bacterial growth (**c**). The *t*-test indicated that the relationship between siderophore production and bacterial growth in the experiment with medium type and mineral type was not significantly (ns) correlated, *p*-value > 0.05.

When strain QCS59 was allowed to grow on three of the most common media (Kings B, succinate, and glucose medium) cited in previous studies, it was found that Kings B was the optimal medium, with a 22.13-fold increase in amount when compared with the succinate medium and a 2.51-fold increase when compared with the glucose medium (Figure 3a). To study their effect on production, several minerals including MgSO_4_·7H_2_O, FeCl_3_, ZnCl_2_, CuSO_4_, CaCl_2_, CdCl_2_, and CoCl_2_ were introduced into the siderophore production medium separately at a 20.0 μM concentration (Figure 3b). All tested minerals were stimulatory compared with the control treatment; the maximum production was attained with CaCl_2_, followed by FeCl_3_ and MgSO_4_·7H_2_O; and CoCl_2_ was the least preferred mineral. Concerning bacterial growth, it reached the highest values with FeCl_3_, while other minerals were inhibitory, and the maximum bacterial inhibition occurred in the presence of CuSO_4_. The impact of iron intensity on siderophore secretion was assessed in the range of 0.0–50.0 μM. The optimal production was obtained at a 10.0 μM concentration, while the optimal growth of strain QCS59 was obtained at 20.0 μM. It was found that siderophore production decreased gradually with rising iron supplementation; therefore, 10 μM was recognized as the threshold level (Figure 3c). The effect of the concentration of the other metals is shown in Figure S1.

**Figure 4:**
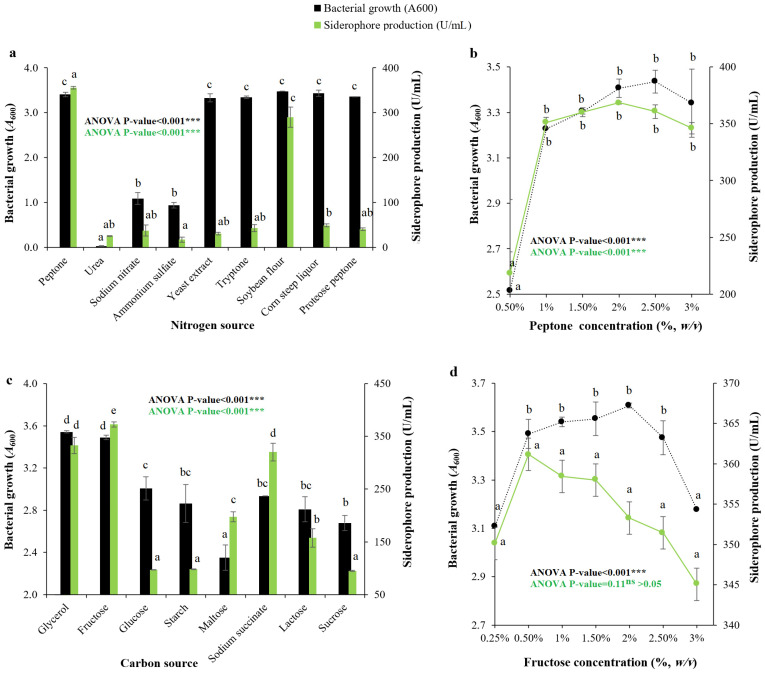
Influence of nitrogen and carbon sources on siderophore production and bacterial growth of *Pseudomonas* sp. QCS59. Nitrogen sources were tested at 1.0% (*w*/*v*) molar content concentration (**a**); these replaced peptone, which is the original nitrogen source used in Kings B. The inoculated replicates were then incubated under optimized conditions. The effect of the nitrogen concentration of the selected source was then tested at 0.5–3.0% (*w*/*v*) (**b**). Carbon sources were examined at 0.5% (*w*/*v*) (**c**); these replaced glycerol, which is the original carbon source in Kings B. Incubation was then performed under the optimized conditions. The effect of carbon source concentration to determine the optimal carbon was then carried out at 0.25–3.0% (*w*/*v*) (**d**). The *t*-test indicated that the relationship between bacterial growth and siderophore production in the experiments with nitrogen and carbon sources was not significantly (ns) correlated, *p*-value > 0.05.

The influence of the nitrogen sources was initially assessed at a 1.0% (*w*/*v*) molar concentration, and they were separately applied to the production medium to study their effect on the siderophore production by *Pseudomonas* sp. QCS59. The maximum value was obtained with peptone, followed by soybean flour. While ammonium sulfate was found to be the least preferred nitrogen source for production (Figure 4a). The influence of peptone concentration on production was then studied, and it was maximal in the range of 1.0–2.5%, with an optimal value obtained at a 2.0% (*w*/*v*) concentration (Figure 4b). The effect of the carbon source was initially explored at 0.5% (*w*/*v*), and it was also introduced into the siderophore production medium separately to study its effect on production. Optimal production was obtained with 0.5% (*w*/*v*) fructose, followed by 0.5% (*v*/*v*) glycerol, while sucrose was the least preferred carbon source (Figure 4c). The effect of fructose concentration on production was then studied (Figure 4d), and it was maximal in the range of 0.5–2.5%, with the optimal value obtained at 0.5% (*w*/*v*) fructose, while the bacterial growth was optimal at a 2.0% (*w*/*v*) concentration.

**Figure 5:**
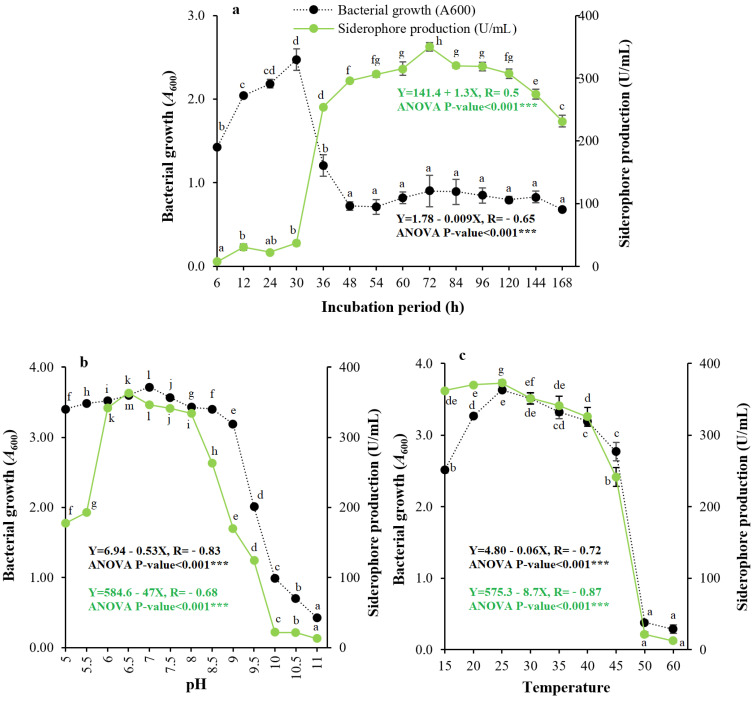
Effects of incubation period (**a**), pH (**b**), and temperature (**c**) on bacterial growth and siderophore production by strain QCS59. The influence of incubation period was tested by growing the selected isolate in the basal medium for various durations (6–168 h). The influence of pH was determined by growing the strain at initial pH values of 5.0 to 11.0, and the effect of incubation temperature was examined at 15–60 °C under the optimized conditions. In all cases, active inocula were made by suspending actively growing colonies of the selected isolate in Luria–Bertani (LB) broth and incubating overnight at 30 °C. The number of bacterial cells per milliliter of inoculum was adjusted nephelometrically at 1 × 10^9^ CFU/mL.

**Figure 6:**
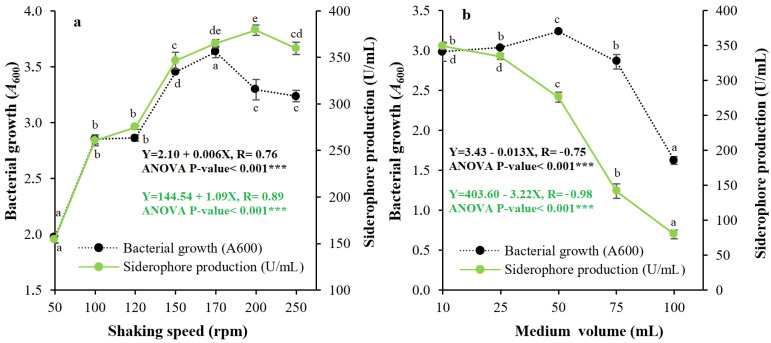
Effects of shaking speed (**a**) and medium volume (**b**) on growth and siderophore production by strain QCS59. Different shaking speeds (50–250 rpm) were tested after inoculating Kings B broth with the tested bacterium and incubating under optimized conditions. The influence of medium volume was tested by growing isolate QCS59 in the modified siderophore production medium in 125 mL conical flasks with different medium volumes (10–100 mL). At the end of every experiment, siderophore production and growth were assayed according to the standard procedures mentioned above.

Regarding the growth phase of siderophore, production was found to fluctuate with incubation time (6–168 h). The maximum amount of siderophore produced from strain QCS59 was observed after 72 h of incubation after bacterial growth presented a maximum at 30 h of incubation (Figure 5a). Figure 5b demonstrates the capability of isolate QCS59 to produce siderophore over a wide range of pH (6.0 to 8.0), with the optimal value being pH 6.5. Incubation temperature likewise had an essential role in siderophore production; strain QCS59 was grown in Kings B medium at temperatures of 15–60 °C. It produced the maximal amount of PVD over a wide spectrum of temperatures (15–40 °C), with the maximal value at 25 °C (Figure 5c). Figure 6a shows the effect of shaking speed on production, and the maximum amount was observed at 200 rpm. The results in Figure 6b show the effect of medium volume (10.0–100.0 mL) in a 125 mL conical flask = on production. The maximal level of siderophore was observed at a 10.0 mL medium volume (lowest medium volume and highest amount of aeration). 

**Figure 7:**
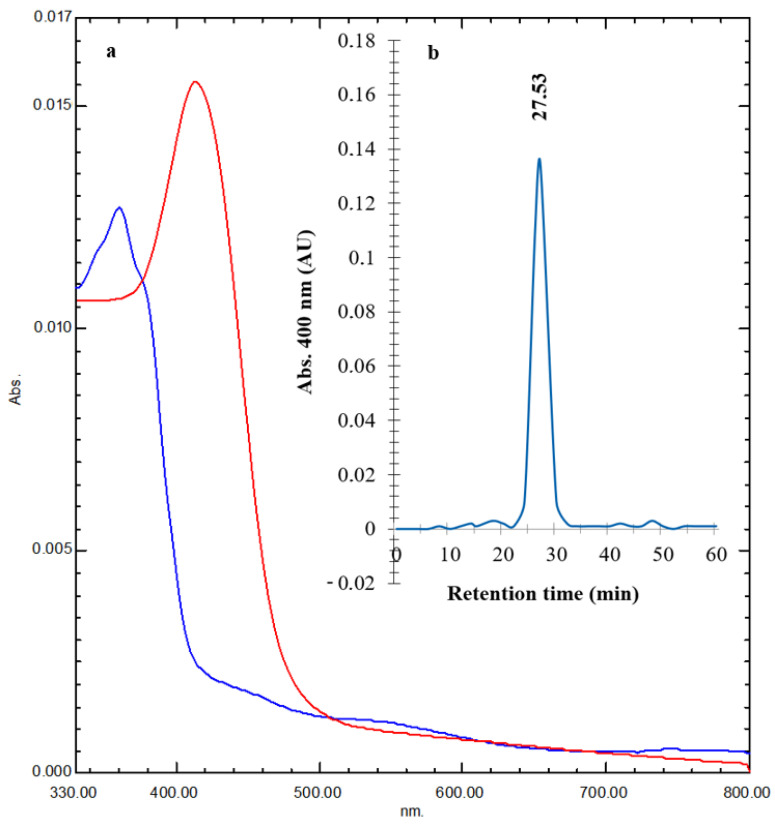
Panel (**a**) shows the UV spectra of both the acidified PVD (pH 3.0, red line) and the neutral PVD (pH 7.0, blue line) recovered from a gel permeation column packed with Sephadex G-25 with dimensions of 1.5 × 100 cm^2^. The gel was pre-equilibrated with distilled water, and the elution was performed with distilled water at a low flow rate of 0.3 mL/min. Three-milliliter fractions were pooled, and the PVD-containing fractions were pooled. UV spectral analysis was performed using a UV–Vis spectrophotometer for both the acidified and the neutral forms of PVD at pH 3.0 and 7.0, respectively. The purified PVD was lyophilized and stored at 4 °C for the next experiments. Panel (**b**) represents the HPLC–Vis chromatogram of the refined siderophore examined via RP-HPLC at *A*_400_.

The tested PVD was refined in two main steps: chelation cation exchange chromatography and gel permeation chromatography through Sepharose FF resin and Sephadex G-15 resin, respectively. The fluorescence of PVD was pH-dependent. Below pH 7, it almost lost its characteristic green color, while at pH 7.0 and higher, it conserved its characteristic color. At low pH (3.0), the absorbance presented a maximum at 360 nm (red line), while the neutral form presented a maximum at 414 nm (blue line) (Figure 7a). To reveal the type of PVD purified from strain QCS59, fractions showing detectable absorbance at *A*_400_ were investigated using RP-HPLC. A major peak was found at an RT of 27.53 min, verifying its purity (Figure 7b).

### 2.3. Thermal and Storage Stability of QCS59 Siderophore

**Figure 8:**
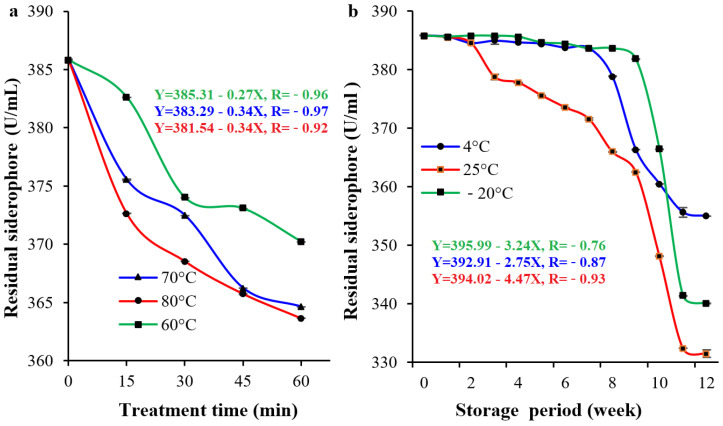
Effect of elevated temperature (**a**) and storage temperature (**b**) on the stability of QCS59 siderophore. Thermal stability was tested with a 60 min exposure to high temperatures (60–80 °C). The stability was checked in 15 min intervals by measuring the residual siderophore level. In addition, the storage stability of siderophore was evaluated by storing a preparation at the most common storage temperatures, 4 °C, −20 °C, and 25 °C, for three months. Weekly measurements of residual siderophore levels were performed. Two-way analysis of variance (ANOVA) showed that both high temperature and long storage had significant consequences on siderophore, *p*-values < 0.001.

Various thermal treatments were studied and are represented graphically in Figure 8a. The thermal treatment of siderophore followed a regression pattern with increasing temperature and exposure time. By the end of experiment, the tested siderophore retained most of its initial values (385.84 U/mL). It retained 96.5%, 94.5%, and 94.3% of its initial value at 60 °C, 70 °C, and 80 °C, respectively. Various storage temperatures over long periods were studied and are represented graphically in Figure 8b. The optimal storage stability after 3 months occurred at −20 °C, followed by 4 °C, while the storage at 25 °C resulted in the lowest stability. At −20 °C, the stability of siderophore was the highest during the first 8 weeks. By the completion of the 12th week, 92% of the siderophore remained. At 4 °C, the stability was nearly unchanged by the end of the ninth week of storage. By the completion of the 12th week, the remaining siderophore level was 88.1% of the initial value. At 25 °C, the stability of siderophore was the highest from 1 to 4 weeks of storage. By the completion of the 12th week, 85.1% of the initial siderophore remained.

### 2.4. Antibacterial Activity of QCS59 Siderophore

**Figure 9:**
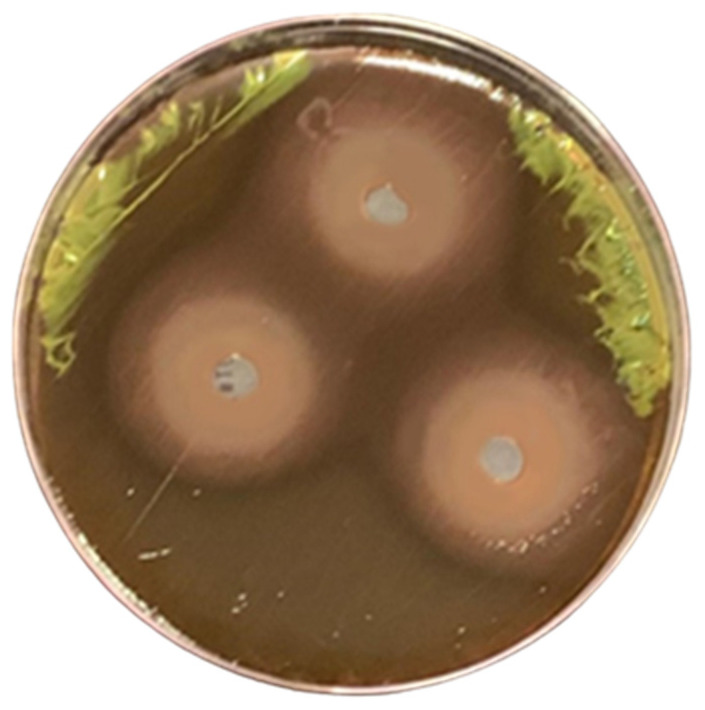
Antibacterial activity of QCS59 siderophore against *E. coli* ATCC 8739 grown on eosin methylene blue medium. The bacterial growth was drastically inhibited, and almost all the characteristic green metallic sheen disappeared. A total of 40 µL (18.75 µg) of siderophore preparation was applied into each 8 mm well and incubated for 20 h at 37 °C.

While examining the effect of siderophore on many pathogenic bacteria, different inhibitory and/or killing levels were noted (Table 1). The most affected bacterium was *E. coli* ATCC 8739 (34.3 mm inhibition zone with an MIC of 6.25 µg/mL and MBC of 12.5 µg/mL) (Table 1, Figure 9). This was followed by *Enterobacter aerogenes* ATCC 13048 (MIC of 25.0 µg/mL and MBC of 50.0 µg/mL). The lowest antibacterial action was found against *P. aeruginosa* ATCC 27853 (MIC of 200 µg/mL and MBC of >400.0 µg/mL). Furthermore, the MIC against *S. enterica* ATCC 13076 and *Shigella flexneri* ATCC 12022 was 50 µg/mL; however, the MBC against *S. enterica* ATCC 13076 was lower (100.0 µg/mL).

### 2.5. Antibiofilm Activity

**Figure 10:**
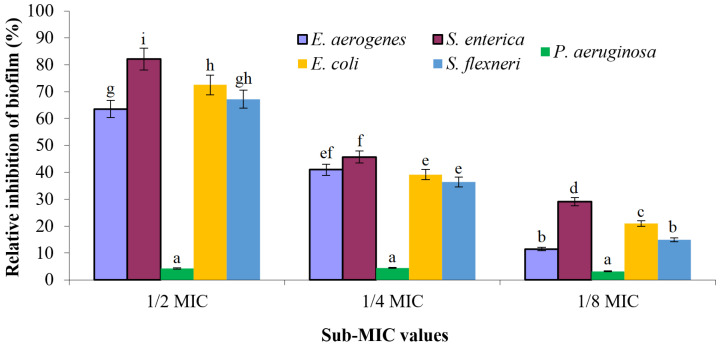
Antibiofilm activity of siderophore at sub-MIC values against several pathogenic bacteria.

The percentages of biofilm inhibition directly correlated with the siderophore concentration (Figure 10). The treatment of *E. aerogenes*, *S. enterica*, *P. aeruginosa*, *E. coli*, and *S. flexneri* biofilms with 1/2 MIC values of siderophore were 63.5%, 82.1%, 4.2%, 72.5%, and 67.2%, respectively, compared to that of the control. The 1/4 MIC values of siderophore inhibited the biofilms formation to 41.0%, 45.7%, 4.5%, 39.2%, and 36.4%, respectively. The lowest inhibition of biofilm formation was noticed when treating the cells with 1/8 of the MIC value of siderophore, in which percentages of biofilms inhibition were 11.5%, 29.1%, 3.2%, 21.0%, and 14.9%, respectively. It is evident that maximal biofilm inhibition occurred for *S. enterica*, and no significant changes in the adherent biofilms were noticed in case of *P. aeruginosa* compared to the control.

### 2.6. Oxidative Stress and Lipid Peroxidation

The oxidative stress in the treated bacteria against the blanks was evaluated by assessing the activity of the SOD and CAT antioxidative enzymes. Furthermore, the release of MDA because of the lipid peroxidation of cell membranes was assessed. Compared with CAT, there was no significant difference in the enzymatic activity of SOD between treated and nontreated cells for any of the bacteria after exposure to siderophore. The change in SOD level was in this order: *E. coli* (1.56-fold) > *E. aerogenes* (1.40-fold) > *S. enterica* (1.17-fold) > *S. flexneri* (1.15-fold) > *P. aeruginosa* (1.07-fold). However, significant changes in CAT and MDA for most of the bacteria except *P. aeruginosa* were detected (Table 2). The change in CAT activity occurred in this order: *E. coli* (2.93-fold) > *S. enterica* (2.45-fold) > *E. aerogenes* (2.25-fold) > *S. flexneri* (2.18-fold) > *P. aeruginosa* (1.44-fold) (Table 2). Interestingly, there was a significant rise in MDA after exposure to siderophore; the maximum MDA concentration was found for the treated *E. aerogenes* (9.53-fold) and *E. coli* (8.15-fold), while the lowest was observed for the treated *S. enterica* (4.52-fold), *S. flexneri* (2.38-fold), and *P. aeruginosa* (1.25-fold) (Table 2).

### 2.7. SEM Analysis

**Figure 11:**
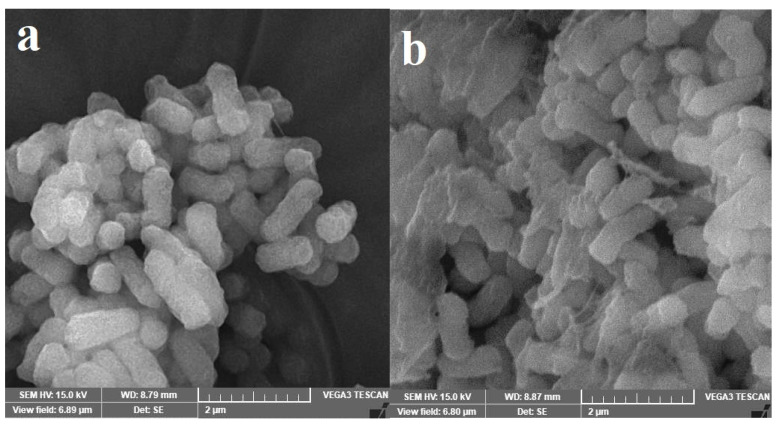

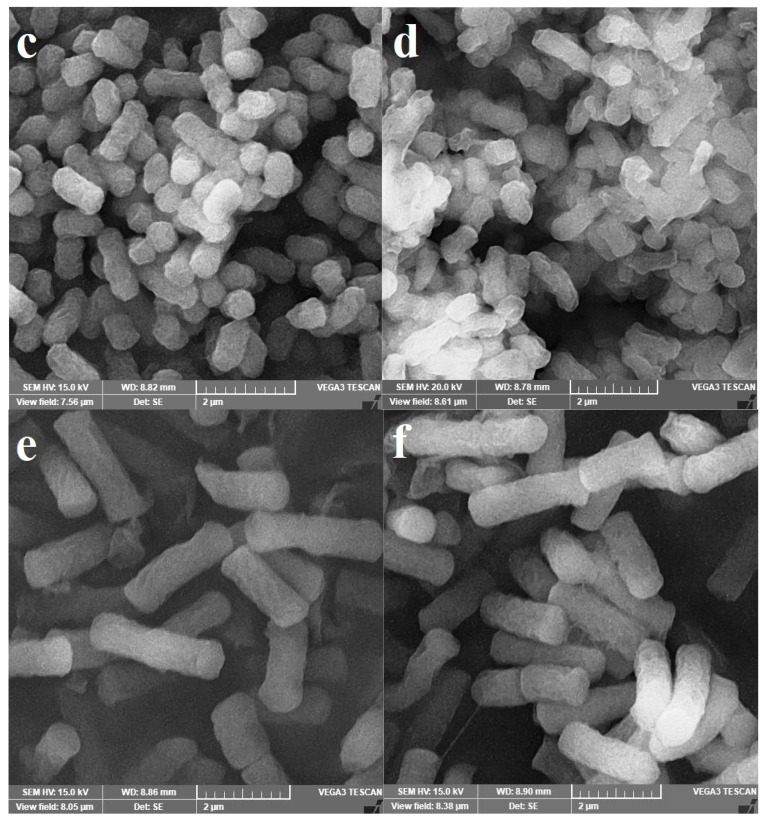

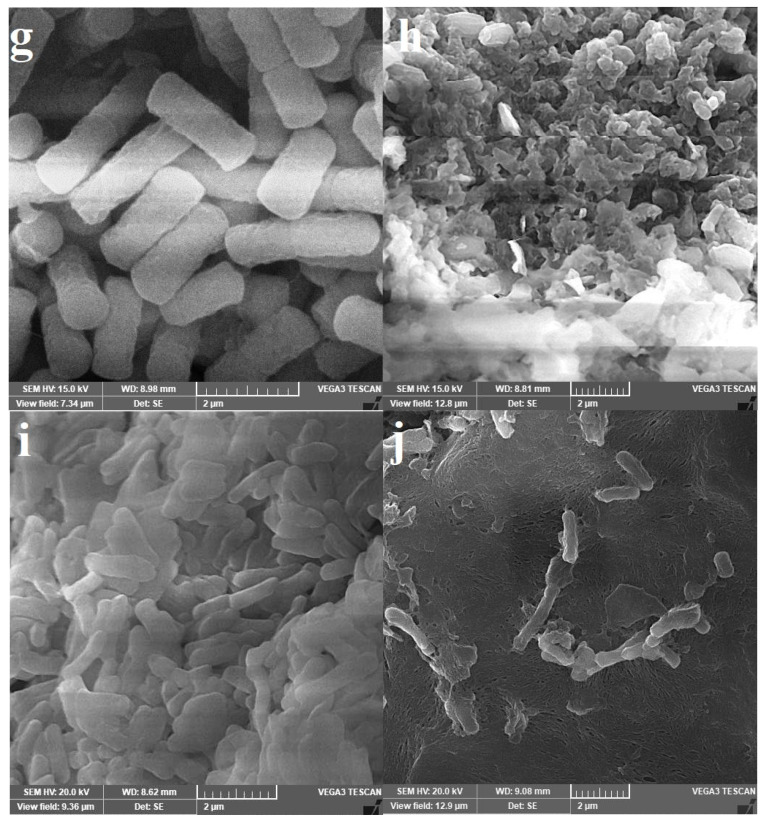
Scanning electron micrographs of *E. coli* ATCC 8739 (**a**,**b**), *S. flexneri* ATCC 12022 (**c**,**d**), *P. aeruginosa* ATCC 27853 (**e**,**f**), *E. aerogenes* ATCC 13048 (**g**,**h**), and *S. enterica* ATCC 13076 (**i**,**j**). The left panels represent the controls, which were treated with physiological saline at 37 °C for 24 h, while the right panels represent the siderophore-treated cells incubated for 24 h at 37 °C. Bacterial cells were fixed, dehydrated with increasing concentrations of ethanol, sputter-coated with chromium, and, finally, the surface morphology of bacterial cells was examined with Vega3 Tescan SEM at low voltages (15–20 kV).

The SEM examination proved that siderophore-treated cells (Figure 11b,d,h,j) suffered variable degrees of shrinkage and surface malformation when compared with the control micrographs (Figure 11a,c,g,i). The highest malformation was observed for *E. aerogenes* ATCC 13048 (Figure 11g,h), and the most cellular damage and lysis were observed for *S. enterica* ATCC 13076 (Figure 11i,j). However, the weakest structural effect was observed for *P. aeruginosa* ATCC 27853 (Figure 11e,f) followed by *S. flexneri* ATCC 12022 (Figure 11c,d).

## 3. Discussion

Siderophores are chiefly produced by microorganisms for the acquisition of iron from the external medium; however, they have other functions including the biocontrol of phytopathogenic fungi and the enhancement in plant growth, and in environmental applications and medical applications as antimalarials, antimicrobials, vaccines, anticancer agents, and drug-delivery mediators [10,17]. The commonly used siderophores are PVDs of *P. fluorescens* SBW25 [18], schizokinen of *Rhizobium leguminosarum* IARI 917 [19], protochelin of *Azotobacter vinelandii* [20], and rhizobactin of *Rhizobium meliloti* [21].

Many studies have emphasized the importance of producing siderophores especially from *Pseudomonas* spp.; unfortunately, full characterization was not conducted, and antibacterial activity at the ultrastructural level was not completely determined. In the present study, a total of sixty-eight fluorescent pseudomonads were isolated from environmental and clinical samples gathered from the eastern region of Saudi Arabia. The highest amount of siderophore was produced by isolate No. 59, which was recovered from the rhizosphere of *H. schimperi*, a plant growing on a farm located in Qatif region. It was genetically identified as *Pseudomonas* sp. QCS59 and given the accession number MT153203 in the GenBank database.

To optimize siderophore production by strain QCS59, many nutritional and physical factors were studied. Nutritional conditions were initially optimized. The effect of medium type is one of the key features studied in the literature. The maximum siderophore secretion was found when using Kings B (Figure 1c). This could be attributed to the richness of Kings B in peptone, which includes several proteins, amino acids, and some elements such as calcium, magnesium, potassium, and sodium. The preference for Kings B for the induction of production agrees with the work of Kotb et al. on *P. aeruginosa* strain ryn32 [16]. This is different from the findings of other studies, where succinate medium was found to be optimal for *P. aeruginosa* marine isolate [22], *P. aeruginosa* Mgrv7 [10], *P. fluorescens* P5-18 [23], and *Pseudomonas strains* GRP3A and PRS [24]. GSA medium induced the highest siderophore production by *Pseudomonas* sp. ANT_H12B [25].

Metal ions were proven to affect siderophore production as they are cofactors for many cellular enzymes. As such, several minerals were introduced separately into Kings B medium at a 20.0 μM concentration. All of them except CoCl_2_ stimulated production as compared with the control. The superiority of metals could be arranged as Ca^2+^, Fe^3+^, Mg^2+^, Zn^2+^, Cu^2+^, and Cd^2+^, respectively. The superiority of Ca^2+^ ions for production coincides with results for *P. aeruginosa* ryn32 [16].

Our results showed that levels of iron as low as 10.0 μM were the best for maximum production (333.82 U/mL). Concentrations above this threshold level suppressed the production process progressively. The reason for this suppression may have been the negative transcriptional control by fur protein where Fe^2+^ ions work as a corepressor. These lower concentrations of iron were also found for siderophore production in *P. aeruginosa* Mgrv7 (15 µM [10]), *P. aeruginosa* FP6 (5 µM [26]), *Pseudomonas* sp. strain PB19 (20 µM [27]), *P. aeruginosa* strain ryn32 (25 µM [16]), and *Pseudomonas* strain GRP3A [24].

Siderophore production is largely influenced by the constituents of the production medium. When studying the effect of several nitrogen sources on the tested strain, the maximum level was obtained at 2% (*w*/*v*) peptone. For bacterial growth, the maximum value was obtained with soybean flour, which could be attributed to the richness of soybean flour in iron, which is a principal element required for bacterial growth and is a repressor for siderophore production. Regarding previous studies, the maximum siderophore production was obtained with ammonium sulfate for *P. aeruginosa* strain ryn32 [16], and urea for *P. aeruginosa* FP6 [26] and *Pseudomonas* sp. strain PB19 [27].

The highest siderophore production by strain QCS59 was obtained by supplementation with 0.5% (*w*/*v*) fructose as the carbon source, while the maximum bacterial growth was noted with 0.5% (*w*/*v*) glycerol. This may have been due to the preference of simple monosaccharides as readily available carbons for production. Regarding previous studies, glucose was optimal for *Pseudomonas* sp. strain PB19 [27], while sucrose and mannitol were optimal for *P. aeruginosa* FP6 [26].

It was also essential to find out the optimum physical conditions affecting siderophore production. These data are required by other investigators and biotechnologists for the upstream production procedure to increase the efficiency and performance of the production system, enhance economic benefits, improve the method, and increase the quality of output. In examining the effect of production period, the highest level of siderophore production was observed at 72 h after bacterial growth, which presented a maximum at 30 h. Optimal production is reached during the stage when bacterial growth declines; this indicates an opposite correlation between bacterial growth and siderophore production, which is a property of the secondary metabolites secreted by bacteria. This coincides with the siderophore produced by *Pseudomonas* strains GRP3A and PRS [24] but is different from that of *P. aeruginosa* ryn32 (60 h [16]) and *P. aeruginosa* Mgrv7 (60 h [10]).

The pH value of a medium plays a key function in siderophore production by affecting the metabolism of bacterial cells and the transport of several nutrient components through plasma membranes [16]. In the current study, the maximum amount of siderophore was attained at a pH range of 6–8, with a peak at pH 6.5. This is of particular importance in the environment of these bacteria as iron is insoluble at pH 6.5 and is not available to the cells in this environment. This limitation of free iron generates siderophore biosynthesis by harboring bacteria. This coincides with the findings of a previous study, where pH 5–8 was optimal for *Pseudomonas* sp. ANT_H12B [25]. In addition, pH 6.0 was best for a marine strain of *P. aeruginosa* [22]. pH 7 was best for *P. aeruginosa* FP6 [26], *P. putida* NCIM 2847, *P. aeruginosa* [28], *P. aeruginosa* Mgrv7 [10], and *Pseudomonas* sp. strain PB19 [27]. Furthermore, pH 7.5 was optimal for *P. aeruginosa* ryn32 [16].

Temperature is an essential factor that should be optimized to maximize siderophore production. The optimal production by the strain under study was found at 25 °C. This coincides with that of *P. aeruginosa* (27.80 °C [28]) and *Pseudomonas* sp. strain PB19 (29 °C [27]); however, 35 °C was optimal for siderophore production by *P. aeruginosa* ryn32 [16] and *P. aeruginosa* Mgrv7 [10]. In addition, the maximum amount of QCS59 siderophore was found at a 200 rpm shaking speed and a 10.0 mL medium volume in 125.0 mL conical flasks. The superiority of higher shaking speeds and lower medium volume might be due to the better homogenization of nutrients and cells, as well as to the better aeration as more oxygen is dissolved in the medium [29,30].

The siderophore was purified, and the absorption peaks were confirmed. The acidified preparation at pH 3.0 produced the maximum absorption at 360 nm, while the neutral preparation at pH 7.0 presented a maximum at 414 nm. These results are consistent with former results [4]; however, the acidic and neutral forms of Mgrv7 siderophore had maximal absorptions at 400 nm and 360 nm, respectively [10]. To verify its homogeneity, the final purified siderophore was analyzed by HPLC with a C18 reverse phase column. This analysis is also useful for RT determination, which allows the verification of typical and atypical PVDs produced by the *Pseudomonas* genus [31]. One major peak was found at an RT of 27.53 min, verifying the purity of QCS59 siderophore. This value coincides with the RT value of the atypical siderophore Pa C (28.04 min, ΔRT 0.51 min). However, it is different from that of the siderophore of another strain of *P. aeruginosa* (22.6 min, ΔRT 4.93 min) [31], the typical PVD-PAO1 (22.5 min, ΔRT 5.03 min), the PVD of *P. fuscovaginae* UPB 1023 (22.10 min, ΔRT 5.43 min), the atypical PVD Pa B (19.07 min, ΔRT 8.46 min), the PVD of *P. aeruginosa* Mgrv7 (18.95 min, ΔRT 8.58 min) [10], Pa A (18.78 min, ΔRT 8.75 min), and the siderophore from an isolate of *Pseudomonas* sp. recovered from chickpea rhizosphere (18.44 min, ΔRT 9.09 min) [32]. The results of the UV spectra and the visible variation in the color of PVD-QCS59 at pH 7.0 and 3.0 together specified that it is an atypical type of PVD siderophore [33,34].

The influence of some factors on the stability of QCS59 siderophore was also studied. The analysis of its thermal stability for 60 min showed that the remaining siderophore at 60 °C was 96.47%, at 70 °C was 94.50%, and at 80 °C was 94.25%. It appeared that siderophore was not affected greatly and had inherent resistance and stability against elevated temperatures due to its cyclic structure, nonprotein nature, and being produced by an endogenous bacterium residing in a harsh environment such as the climate in Saudi Arabia [10,35].

An analysis of the effect of QCS59 siderophore against various pathogenic bacteria such as *S. enterica* ATCC 13076, *P. aeruginosa* ATCC 27853, *S. flexneri* ATCC 12022, *E. coli* ATCC 8739, and *E. aerogenes* ATCC 13048 revealed diverse inhibitory levels. The most affected bacterium was *E. coli* ATCC 8739 with an MIC of 6.25 µg/mL and an MBC of 12.5 µg/mL. The lowest antibacterial action was found against *P. aeruginosa* ATCC 27853 with an MIC of 200 µg/mL and an MBC of more than 400.0 µg/mL. The lowest effect observed against *P. aeruginosa* ATCC 27853 may have been due to the ability of *Pseudomonas* to exploit xenosiderophores as self-molecules [10]. The morphological examination of the treated cells demonstrated shrinkage and surface malformation compared with the control treatments. This may have been due to the cellular deprivation of free heme and other cations because of the coupling with PVD-QCS59 siderophore and/or the complexation of wall and membrane cations with siderophore. This explanation was confirmed upon supplementation of the growing medium with more iron. The antibacterial potential of siderophore decreased in this condition because iron was no longer a growth-limiting factor, and free iron became available for cells; thus, pathogen inhibition did not proceed. Indeed, we observed that the inhibition of bacteria diminished progressively with iron supplementation above 80 µM and completely stalled at 320 µM [13,36].

Furthermore, the sub-MIC values of PVD-QCS59 siderophore were found to have antibiofilm activity in a concentration-dependent manner against most of the tested pathogens. Using the same sub-MIC level, the siderophore significantly inhibited *S. enterica*, and no significant changes in the adherent biofilms were noticed for *P. aeruginosa*. This observation was confirmed by the SEM images. The weakest effect against *P. aeruginosa* may have been due to the ability of this bacterium to utilize xenosiderophores, as reported earlier. The ability of siderophores compared with that of antibiotics to inhibit the biofilm formed by the bacterium was previously reported [37]. In many cases, pathogenic bacteria were found to produce an extracellular polymeric matrix after exposure to antibiotics as a protective shield and proceed in some degree of metabolic dormancy. This makes classical antibiotics less effective; as a result, there has been significant interest among researchers working on materials combating biofilms. While there is some elementary evidence suggesting a relationship between iron acquisition and biofilm formation, the specifics of this connection remain uncertain. In certain pathogens, this link has been well established, but, in many instances, the available literature is limited and inconsistent. Siderophores may inhibit biofilm formation by altering the competition dynamics or by providing signals that may affect the quorum-sensing pathways of those bacteria. They may induce changes in gene expression or metabolic pathways in foreign bacteria, influencing motility, adhesion, and biofilm matrix production [37].

Regarding other siderophores, the pyochelin siderophore of *Burkholderia paludism* greatly inhibited the growth and biofilm formation in many enterococci and staphylococci; the MIC and MBC values against several strains of *E. faecalis* were 3.1 µg/mL and 6.3 µg/mL, respectively. However, the MIC and MBC against several strains of *S. aureus* were 6.3 µg/mL and 25.0 µg/mL, respectively [3]. The Mgrv7 siderophore of *P. aeruginosa* Mgrv7 greatly affected *Candida* spp., especially *C. tropicalis* and *C. albicans*; the MIC and minimum fungicidal concentration (MFC) were ≤128 µg/mL [10]. Furthermore, a siderophore of *Brevibacillus brevis* GZDF3 was synergistic with antibiotics such as amphotericin B against *C. albicans* [38]. The MFC of *P. aeruginosa* siderophore against *A. fumigatus* and *C. albicans* was ≥100 µg/mL, and lower concentrations stalled the dimorphism in *Candida* [39]. The siderophores of *P. putida* showed an outstanding inhibitory action against several fungi [12]. The siderophores produced by *P. fluorescens* MPF47 showed effective inhibitory action against *R. solani* [15]. The siderophores of *P. fluorescens* BBc6R8 showed biocontrol activity against the bacterium *Streptomyces ambofaciens* ATCC23877 [13]. In addition, the siderophore of *P. syringae* BAF.1 has shown potential against *F. oxysporum* [40]. The MFC for *E. coli* catecholate siderophore against *A. nidulans* was 54 µg/mL, and, at lower levels, it induced morphological changes in the normal pattern of the organism [14]. A siderophore of *Alcaligenes feacalis* exerted biocontrol effects against *F. oxysporum* NCIM1008 [41]. These results collectively explain the ability of siderophores as antimicrobial agents; this activity may be a secondary function for the producing microorganisms in the environment in addition to the acquisition of iron and other minerals [8,13].

The exposure of all bacteria except *P. aeruginosa* ATCC 27853 to PVD-QCS59 as a xenosiderophore led to the depletion of the available iron perhaps due to complexation with siderophores outside cells and/or coupling of wall cations with siderophores [42]. This iron-starved status might interrupt the transmembrane potential and ATP generation because iron is a crucial prosthetic element in the structure of oxidative phosphorylation enzymes. The depletion in ATP adversely affects cellular functions, especially the transmembrane potential and selective permeability [43]. On the other hand, some studies attributed the cell death after exposure to siderophores to the ferroptosis mechanism, where siderophores assist in the imbalanced hyperinternalization of iron, perhaps via MirA receptors [6]. Metal stress can shift the equilibrium of ROS homeostasis, where high levels of H_2_O_2_ are generated, which in turn produces hydroxyl ions (OH^−^) and hydroxyl radicals (OH^•^). These can oxidize the cellular macromolecules, including DNA, lipids, RNA, and proteins [5].

To counteract this stress, bacteria employ a defensive antioxidative system involving the SOD enzyme, which could break superoxides into H_2_O_2_, which is further hydrolyzed into H_2_O and O_2_ by CAT [14]. Unfortunately, the overproduction of ROS negatively affects the morphological structures of bacteria as it frequently results in the lipid peroxidation of cell membranes and therefore loss consistency, growth inhibition, and death [15]. The increase in MDA level especially in the case of *E. coli* ATCC 8739 supports this opinion (this was found in the SEM experiment). Comparable rises in CAT, SOD, and MDA have been identified in many microorganisms upon metal stress [44]. It was found that a copper- and zinc-containing SOD is crucial for *C. albicans* to stabilize the negative effects of many oxidative stresses. ROS was found to disable the [4Fe-4S] cluster-containing enzymes via the oxidation and release of iron from the cluster. The released iron can crosslink with H_2_O_2_ to produce OH^−^ radicals [14]. As such, the application of such siderophores in the suppression and biocontrol of wastewater bacteria may be a strategy in addition to the anti-infection strategies that are less likely to generate bacterial resistance. They indirectly kill bacterial cells via the mediation of iron starvation or hyperaccumulation rather than directly affecting the bacterial cell targets [2,9].

## 4. Materials and Methods

### 4.1. Chemicals and Reagents

CAS, hexadecyl trimethyl ammonium bromide (HDTMA), cetrimide agar, nutrient broth, nutrient agar, Muller Hinton Broth (MHB), Muller Hinton Agar (MHA), LB broth, CAT assay kit, lipid peroxidation (MDA) assay kit, and SOD assay kit were obtained from Sigma-Aldrich (Saint Louis, MO, USA). Additional analytical chemicals were bought from local providers. All media in this study, unless otherwise stated, were sterilized for 15 min at 121 °C. CAS reagent was made from two solutions: (1) 60.5 mg of CAS added to 50.0 mL of distilled water, combined with 10.0 mL of 1.0 mM iron chloride hexahydrate in 10.0 mM HCl; (2) 72.9 mg of HDTMA dissolved in 40.0 mL distilled water. After the reagents were dissolved, the later solution was mixed with the first solution, and the pH was adjusted to 7.0 (Mettler Toledo pH meter, Greifensee, Switzerland). CAS agar was prepared by combining 900 mL of sterile nutrient agar and 100 mL of sterile CAS reagent.

### 4.2. Isolation of Siderophore-Producing Pseudomonads

Thirty-three soil samples were gathered from several areas in the eastern region of Saudi Arabia, including Dammam, Anak, Saihat, Khafji, Alhassa, Qatif, Khobar, Jubail, Dhahran, Abqaiq, and Ras Alkhair. Isolation was performed on the specific medium cetrimide agar, with incubation at 30 °C for 48 h, to allow the growth of suspected *Pseudomonas* spp. In addition, thirty-eight clinical isolates were obtained from local hospitals. Recovered pseudomonads were maintained at −80 °C in 20% sterile glycerol until the next investigations.

### 4.3. Production and Assay of Siderophore

Exactly 1 mL of actively growing cells was inoculated into a 100.0 mL flask holding 25 mL of Kings B broth at pH 7.0. The overnight-grown inocula were prepared in LB broth comprising (g/L) yeast extract (5.0), NaCl (5.0), and tryptone (10.0). The number of bacterial cells per milliliter inoculum was adjusted nephelometrically to 1 × 10^9^ colony-forming units/mL. Incubation was allowed for 72 h at 25 °C and 150 rpm, then siderophores were quantified in culture filtrates after centrifugation for 15 min at 5000 rpm (Hettich Centrifuge REF: 1406, Westphalian, Germany).

Siderophore assays are very sensitive to the presence of metals. As such, before assays, all glassware was left in 6 M HCl for 24 h, then washed thoroughly with deionized water to remove all the remaining metals, especially iron. The qualitative assay was carried out by spotting actively growing bacterial isolates on the surface of CAS agar, with incubation for 48 h at 25 °C. The appearance of orange zones confirmed siderophore production. A quantitative assay of siderophore was conducted at *A*_400_, with each unit (U/mL) expressed as the quantity of siderophore that caused a 0.01 increase in absorbance using a UV–Vis spectrophotometer (UV5 Mettler Toledo, Greifensee, Switzerland) [16]. The control was made from noninoculated culture supernatant.

Siderophore + Fe^3+^-dye (blue) → dye (orange) + Fe^3+^-siderophore complex

### 4.4. Molecular Characterization of the Selected Isolate

Isolate number 59 was identified molecularly by sequencing of the *16SrDNA* gene. Initially, DNA was extracted, then the target gene was amplified with PCR using 27F primer (5′-AGAGTTTGATC(AC)TGGCTCAG-3′) and 1492R primer (5′-CGG(CT)TACCTTGTTACGACTT-3′). Horizontal electrophoresis and sequencing of the PCR product were performed. BLAST analysis was used to search for homologous sequences, which were then deposited into the National Center for Biotechnology Information GenBank library. Finally, a neighbor-joining phylogenetic tree was generated with MEGA11 software.

### 4.5. Optimization of Siderophore Production

Nutritional parameters critically influence the siderophore biosynthesized by bacteria; therefore, the influence of usual media such as succinate, Kings B, and glucose on production was considered at pH 7.0. The Kings B medium consisted of (g/L) K_2_HPO_4_ (1.5), peptone (20.0), MgSO_4_·7H_2_O (1.5), and glycerol (10.0 mL). The glucose medium was composed of (g/L) K_2_HPO_4_ (0.56), glucose (10.0), and urea (0.85). Succinate medium comprised (g/L) (NH_4_)_2_SO_4_ (1.0), succinic acid (4.0), MgSO_4_·7H_2_O (0.2), K_2_HPO_4_ (6.0), and KH_2_PO_4_ (3.0) [11]. Incubation was conducted at 25 °C and 150 rpm for 72 h, and uninoculated culture media were taken as blank.

The optimized medium was then separately supplemented with common minerals (MgSO_4_·7H_2_O, FeCl_3_, ZnCl_2_, CuSO_4_, CaCl_2_, CdCl_2_, and CoCl_2_) at 20.0 μM concentration to determine the optimal minerals for production. Urea, sodium nitrate, ammonium sulfate, yeast extract, tryptone, soybean flour, corn steep liquor, and proteose peptone were tested as nitrogen sources at 1.0% (*w*/*v*) molar content of nitrogen. These replaced peptone, which is the original nitrogen source used in Kings B. The inoculated replicates were then incubated under optimized conditions. The effect of nitrogen source concentration of the selected source was then tested at 0.5–3.0% (*w*/*v*). Fructose, glucose, starch, maltose, sodium succinate, lactose, and sucrose were examined as carbon sources and were applied at the molar content of carbon. These replaced glycerol, which is the original carbon source in Kings B. Incubation was then performed under optimized conditions. The effect of carbon source concentration for the optimal carbon was studied at 0.25–3.0% (*w*/*v*) concentration.

The influence of environmental parameters such as growth phase on the production of siderophore was examined by incubation of the selected isolate in the optimized medium for various periods (6–168 h). The influence of medium pH was examined at pH 5.0–11.0, and the impact of temperature was examined at 15–60 °C under the adjusted parameters. Additionally, shaking speeds of 50–250 rpm and medium volumes of 10.0–100.0 mL in a 125.0 mL flask volume were examined. By the end of the experiments, production and bacterial growth were measured as mentioned above.

### 4.6. Siderophore Purification

Siderophore was purified following the protocol of Xiao and Kisaalita [4] with minor changes. The culture filtrate of *Pseudomonas* sp. QCS59 was adjusted to pH 7.0 with 1 M N-2-hydroxyethylpiperazine-N’-2-ethanesulfonic acid (HEPES) buffer and eluted through a strong cation exchange resin Sepharose Fast Flow (FF) column that was saturated with copper sulfate for chelation of eluted PVDs. The column was 1.5 × 25 cm^2^ (Amersham BioSciences, Buckinghamshire, UK) and was equilibrated with 0.02 M HEPES buffer (pH 7.0) involving 100 mM NaCl at a flow rate of 1.5 mL/min. After elution of PVD, the column was washed with 200 mL of 20 mM acetate buffer (pH 5.0) involving 0.1 M sodium chloride. The PVD-containing fractions were combined, lyophilized, and resuspended in 1 mL of deionized water with 0.01 M EDTA. It was then eluted through a gel permeation column packed with Sephadex G-25 with dimensions of 1.5 × 100 cm^2^. Distilled water was used for both gel equilibration and elution at a low flow rate of 0.3 mL/min. Three-milliliter fractions were collected, and the PVD-containing fractions were combined. The UV spectral analysis using a UV–Vis spectrophotometer (Shimadzu UV-1900, Duisburg, Germany) was performed for both acidified and neutral PVD at pH 3.0 and 7.0, respectively. Siderophore was checked for homogeneity by reverse-phase HPLC (RP-HPLC) (4.6 mm × 100 mm C18 column, Agilent Technologies Inc., Santa Clara, CA, USA). The eluants were system A, composed of 0.1% formic acid, and system B, consisting of 0.1% formic acid and 99.9% acetonitrile. The RT was determined; the purified PVD was lyophilized and kept at 4 °C until the next tests.

### 4.7. Siderophore Stability

The thermal stability of siderophore was tested by 60 min exposure to high temperatures (60–80 °C). The stability was checked in 15 min intervals by measuring the residual siderophore level in the form of Δ*A*_400_. In addition, the storage stability of siderophore was evaluated by storing a preparation at the most common storage temperatures, −20 °C, 4 °C, and 25 °C, for three months. Weekly measurements of residual siderophore levels were performed, and Δ*A*_400_ was calculated.

### 4.8. Assay of Antibacterial Activity 

The MICs of the tested siderophore against *S. flexneri* ATCC 12022, *E. coli* ATCC 8739, *S. enterica* ATCC 13076, *P. aeruginosa* ATCC 27853, and *Enterobacter aerogenes* ATCC 13048 were evaluated via the broth dilution procedure [9] with a few modifications. A 4 mL stock siderophore solution was prepared at 400 µg/mL in MHB, then two-fold dilutions were prepared in the same medium until reaching a concentration of 1.6 µg/mL. Precisely, 200 μL of bacterial suspension in PBS at 1.0 × 10^9^ CFU/mL was used to inoculate each dilution. A broth with no bacterial inoculation was used as the negative control; however, a broth culture without siderophore was used as the positive control (100% growth). The antibiotic ciprofloxacin was used as a standard for comparisons. Incubation continued for 24 h at 37 °C, then the MIC was calculated. The MIC is expressed as the lowest concentration of siderophore suppressing the growth of bacteria. To verify the MBC, 100 µL of various concentrations of siderophore above the MIC levels were subcultured on MHA agar plates. The MBC was identified as the minimal level of siderophore suppressing colony development in petri dishes.

### 4.9. Assay of Antibiofilm Activity

Antibiofilm activity was assessed according to Sandasi et al. [45] and Mohsenipour and Hassanshahian [46], with modifications. The biofilms of the tested bacteria were permitted to establish freely for 24 h before the addition of the tested siderophore. A total of 100 μL of bacterial suspension (*A*_590_ = 0.02 corresponding to 1.0 × 10^6^ CFU/mL) prepared in tryptic soy broth was inoculated into sterile flat-bottomed 96-well microtiter plates. Then, 100 μL of specific siderophore concentrations (1/2, 1/4, and1/8 MIC) was added and incubated for 24 h at 37 °C under static condition. Controls representing 100% adherent biofilms were prepared by incorporating equivalent volume of sterile distilled water instead of siderophore preparation. By the end of incubation, the crystal violet (CV) staining procedure [45] was performed to measure the remaining biofilm biomass. The wells were gently drained, and microtiters were rinsed three times with sterile distilled water to eliminate nonadherent cells. The plates were air-dried and then oven-dried at 60 °C for 45 min. Then, 150 μL of 96% methanol was added to the wells for 20 min to fix the adherent cells. The plates were emptied, and the adhered cells stained with 100 μL of 0.1% CV for 20 min at room temperature. Excess stain was rinsed off by washing the plates at least five times with distilled water. Thereafter, the biofilm biomass was evaluated by resolubilizing the CV bound to the adherent cells with 150 μL of 100% ethanol. The solution was gently shaken, and the *A*_590_ was taken using a microplate reader (BioTek Elx808, Boston, MA, USA). The final results were calculated as shown below [45]:

% biofilm inhibition = [(*A*_490 control_ − *A*_490 sample_)/*A*_490 control_] ×  100

### 4.10. Oxidative Stress and Membrane Peroxidation Assays

After suspending the well-established bacterial grown in siderophore preparations at the MIC value for each bacterium, the endogenous cellular proteins and the stress response enzymes were extracted. This was achieved by suspending one hundred milligrams from freeze-dried cells in lysis buffer. The latter was composed of 25 mM Tris-HCl, 1 mM EDTA, 700 mM β-mercaptoethanol, 1% SDS, and 7 M urea. Cells were disrupted with a homogenizer and centrifuged at 13,500× *g* for 40 min. Initially, the enzymatic activity of SOD was measured by mixing 100 µL of cell-free supernatant with 3 mL of reaction mixture comprising 100 mM sodium phosphate buffer (pH 7.4), 2.25 mM nitroblue tetrazolium chloride, 60 μM riboflavin, 1.5 M sodium carbonate, 30 mM EDTA, and 200 mM methionine. The reaction continued for 10 min at room temperature, and *A*_560_ was measured. The unit of activity is expressed as the amount of SOD required to inhibit 50% of substrate reaction. The specific enzyme activity of SOD is presented as U/mg protein after measuring the total protein content at *A*_280_ [5]. In addition, the enzymatic activity of CAT was evaluated by mixing 50 µL of the cell-free supernatant with 1.5 mL of PBS (pH 7.4) and 950 μL of distilled water. The substrate H_2_O_2_ was prepared by mixing 100 mL of deionized water with 775 μL of 30% (*v*/*v*) H_2_O_2_. The assay started by mixing 500 µL of substrate solution with the reaction mixture. The reaction was directly measured at room temperature, and *A*_240_ was measured. The existence of CAT was monitored by the drop in absorbance at 240 nm (Δ*A*_240_). The specific enzyme activity of CAT is presented as U/mg protein after measuring the total protein content at *A*_280_ [5]. Furthermore, the release of MDA from the peroxidation of membrane lipids was achieved by suspending 0.5 g of cell biomass in 0.1% (*w*/*v*) trichlorohteacetic acid with homogenization and then centrifugation at 21,000× *g* for 15 min. Exactly 1 mL of cell-free supernatant was mixed with 4 mL of 0.5% thiobarbituric acid in 20% trichloroacetic acid while boiling for 30 min at 95 °C, then centrifugation at 150× *g* for 10 min, to measure the absorbance of the supernatant at *A*_532_ and *A*_600_ [5].

### 4.11. Scanning Electron Microscopy (SEM)

The tested microbes were grown in LB broth for 24 h at 37 °C to obtain well-established microbial growth, and microbes were then harvested at 5000 rpm for 15 min. A cell fraction from each bacterium was resuspended in QCS59 siderophore preparation at the respected MIC concentration (µg/mL) for another 24 h at 37 °C. The other cell portion was resuspended in PBS under similar conditions. Cells were harvested and rinsed twice in 200 mM sodium phosphate buffer (pH 7.4) and then suspended for 24 h in a fixative solution composed of 1.5% (*v*/*v*) paraformaldehyde, 1.5% (*v*/*v*) glutaraldehyde, and 2 mM CaCl_2_ in 200 mM sodium phosphate buffer (pH 7.4). Thereafter, the cells were dehydrated with a rising series of aqueous ethanol (20.0, 40.0, 60.0, 80.0, and 99.8%, *v*/*v*) for 15 min each, while a 60 min exposure time was applied for absolute ethanol. The final drying of bacterial preparations was performed by keeping the cells in a drying oven for 2 h at 35 °C. Finally, cells were sputter-coated with chromium, and Tescan Vega3 SEM was operated to study the outer surface morphology at a voltage of less than 10 keV to avoid cellular damage.

### 4.12. Statistical Analysis

If not mentioned elsewhere in this study, all readings were made in triplicate, and the final readings are presented as mean ± standard mean error. Analysis was performed with the statistical package SPSS 29.0. One-way ANOVA followed by Tukey’s honestly significant difference multiple comparisons test were conducted to compare multiple groups. All data were examined for normality and homogeneity of variance by Levene’s and Shapiro–Wilk tests. A *p*-value < 0.05 was considered a significant level. Averages in the same index with different symbols or letters were significantly different (*p*-value < 0.05), where *p* ** < 0.01, and *p* *** < 0.001.

## 5. Conclusions

Based on the obtained results, the siderophore produced by *Pseudomonas* sp. QCS59 recovered from the rhizosphere of *H. schimperi* may be an effective antibacterial agent for the treatment and biocontrol of many enteric bacteria such as *S. enterica*, *E. aerogenes*, *E. coli*, and *S. flexneri*. The maximal amounts of this metabolic product were produced after optimizing the nutritional and physical factors, and low levels (10.0 μM) of iron were best for maximizing production (333.82 U/mL). The purity and homogeneity were confirmed via RP-HPLC and the characteristic peaks that appeared in the chromatogram. The siderophore was thermally stable at high temperatures, possibly due to being produced by an endogenous bacterium residing in a harsh environment. Interestingly, the sub-MIC values had antibiofilm activity against most of the tested pathogens, especially *S. enterica.* There was a rise in MDA and the antioxidative enzymes in treated bacteria because of the peroxidation of membrane lipids and oxidative stress, respectively. The SEM study confirmed the cellular lysis and surface malformation of most of the treated bacteria. The ability of some siderophores such as QCS59 compared with that of antibiotics to inhibit the biofilms formed by pathogenic bacteria has been relatively recently observed. The observation of the ability of many bacteria to protect themselves via the production of extracellular polymeric matrix in the form of biofilms after exposure to antibiotics making them less effective has attracted researchers to find novel antibiofilm agents such as siderophores. While there is some elementary evidence suggesting a relationship between iron acquisition and biofilm formation, the specifics of this connection remain uncertain. It was proposed that siderophores can inhibit biofilm formation by altering the competition dynamics or by providing signals that may affect the quorum-sensing pathways of those bacteria. They may induce changes in gene expression or metabolic pathways in foreign bacteria, influencing motility, adhesion, and biofilm matrix production. Future studies should focus on possible applications in the biocontrol of phytopathogenic fungi, the enhancement in iron uptake by plants, and biocompatibility with different antibiotic and antiseptic formulations.

## Figures and Tables

**Table 1:** Influence of QCS59 siderophore on the growth of numerous pathogenic bacteria.

Tested Species	MIC (µg/mL)	MBC (µg/mL)
*Salmonella enterica* ATCC 13076	50.00	100.0
*Enterobacter aerogenes* ATCC 13048	25.00	50.0
*Escherichia coli* ATCC 8739	6.25	12.5
*Pseudomonas aeruginosa* ATCC 27853	200.00	>400.0
*Shigella flexneri* ATCC 12022	50.00	200.0

The antibacterial activity of siderophore was compared with that of the antibiotic ciprofloxacin. MIC values of the antibiotic were ≥0.50, 2.00, 8.00, 0.25, and 0.02 μg/mL against *S. enterica*, *E. coli*, *S. flexneri*, *P. aeruginosa*, and *E. aerogenes*, respectively.

**Table 2:** Response of antioxidative enzymes and membrane lipid peroxidation after exposure of pathogenic bacteria to QCS59 siderophore.

Tested Species	SOD (U/mg Protein)		CAT (U/min/mg Protein)		MDA (nM/g Biomass)	
	Blank	Treated	Blank	Treated	Blank	Treated
*Salmonella enterica* ATCC 13076	4.6 ± 0.2	5.4 ± 0.4	143.4 ± 11.2	351.0 ± 14.6	31.4 ± 2.0	142.0 ± 12.4
*Enterobacter aerogenes* ATCC 13048	3.7 ± 0.1	5.2 ± 0.3	162.5 ± 9.2	365.0 ± 20.1	17.3 ± 1.0	164.8 ± 15.0
*Pseudomonas aeruginosa* ATCC 27853	3.0 ± 0.4	3.2 ± 0.2	188.0 ± 12.4	271.2 ± 16.8	25.1 ± 2.1	31.5 ± 2.1
*Escherichia coli* ATCC 8739	3.9 ± 0.2	6.1 ± 0.4	154.2 ± 7.0	452.3 ± 30.2	22.5 ± 1.8	183.4 ± 12.5
*Shigella flexneri* ATCC 12022	5.2 ± 0.3	6.0 ± 0.5	176.2 ± 10.2	384.0 ± 25.3	41.5 ± 3.2	98.6 ± 6.8

## Data Availability

The datasets in this research can be found in online repositories. The names of the repositories and accession numbers are included in this article.

## Supplementary Materials

The following supporting information can be downloaded at: https://www.mdpi.com/article/10.3390/ph17091126/s1, Figure S1: Effect of the concentration of different minerals on siderophore production and growth of *Pseudomonas* sp. QCS59.
