# Supplementary material for: Production and Antibacterial Activity of Atypical Siderophore from Pseudomonas sp. QCS59 Recovered from Harpachene schimperi
Source: Pharmaceuticals (Basel). 2024 Aug 26;17(9):1126. doi: 10.3390/ph17091126 (PMC11434927; doi:10.3390/ph17091126)
Supplement: Supplementary file 1 [file pharmaceuticals-17-01126-s001.zip › pharmaceuticals-3152375-supplementary.pdf]

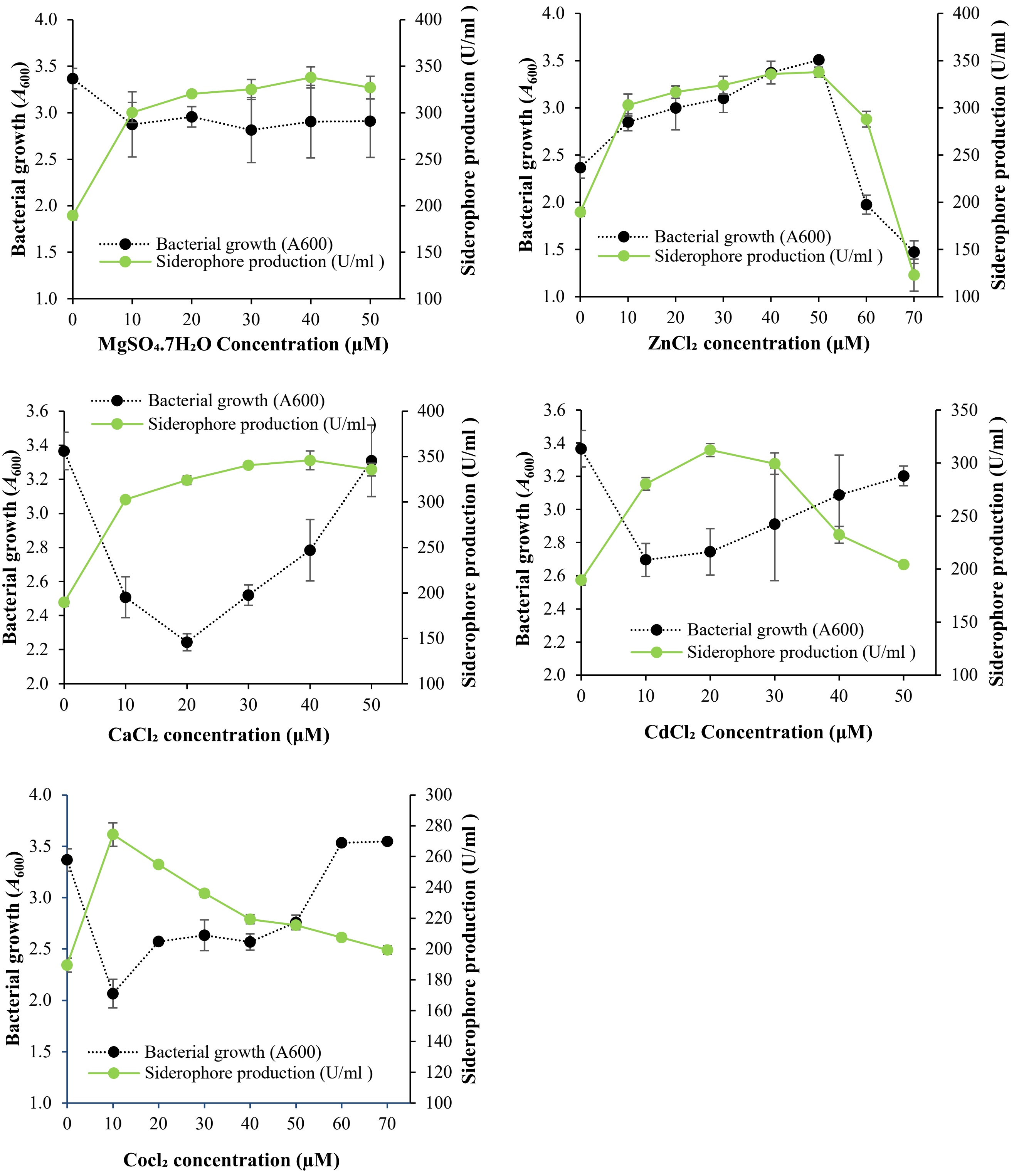

Figure S1. Effect of the concentration of different minerals on siderophore productivity and growth of *Pseudomonas* sp. QCS59.
